# Impact of Macrolide Resistance on Azithromycin for Prevention of Rehospitalization or Death Among Children Discharged From Hospitals in Western Kenya

**DOI:** 10.1093/infdis/jiaf208

**Published:** 2025-04-21

**Authors:** Polycarp Mogeni, John Benjamin Ochieng, Hannah E Atlas, Kirkby D Tickell, Doreen Rwigi, Kevin Kariuki, Laura Riziki Aluoch, Catherine Sonye, Evans Apondi, Lilian Ambila, Mame M Diakhate, Benson O Singa, Jie Liu, James A Platts-Mills, Ferric C Fang, Judd L Walson, Eric R Houpt, Patricia B Pavlinac

**Affiliations:** Center for Microbiology Research, Kenya Medical Research Institute, Nairobi, Kenya; Department of Global Health, University of Washington, Seattle, Washington, USA; Center for Global Health Research, Kenya Medical Research Institute, Kisumu, Kenya; Department of Global Health, University of Washington, Seattle, Washington, USA; Department of Global Health, University of Washington, Seattle, Washington, USA; Center for Microbiology Research, Kenya Medical Research Institute, Nairobi, Kenya; Center for Microbiology Research, Kenya Medical Research Institute, Nairobi, Kenya; Center for Global Health Research, Kenya Medical Research Institute, Kisumu, Kenya; Center for Global Health Research, Kenya Medical Research Institute, Kisumu, Kenya; Center for Global Health Research, Kenya Medical Research Institute, Kisumu, Kenya; Center for Global Health Research, Kenya Medical Research Institute, Kisumu, Kenya; Department of Global Health, University of Washington, Seattle, Washington, USA; Center for Clinical Research, Kenya Medical Research Institute, Nairobi, Kenya; School of Public Health, Qingdao University, Qingdao, China; Division of Infectious Diseases and International Health, Department of Medicine, University of Virginia, Charlottesville, Virginia, USA; Division of Allergy and Infectious Diseases, Department of Medicine, University of Washington, Seattle, Washington, USA; Department of Laboratory Medicine and Pathology, University of Washington, Seattle, Washington, USA; Department of Microbiology, University of Washington, Seattle, Washington, USA; Department of Global Health, University of Washington, Seattle, Washington, USA; The Childhood Acute Illness and Nutrition Network, Nairobi, Kenya; Department of International Health, Johns Hopkins University, Baltimore, Maryland, USA; Department of Medicine, Johns Hopkins University, Baltimore, Maryland, USA; Department of Pediatrics, Johns Hopkins University, Baltimore, Maryland, USA; Division of Infectious Diseases and International Health, Department of Medicine, University of Virginia, Charlottesville, Virginia, USA; Department of Global Health, University of Washington, Seattle, Washington, USA; Department of Epidemiology, University of Washington, Seattle, Washington, USA

**Keywords:** macrolide resistance, azithromycin, hospital, postdischarge, mortality, gut microbiome

## Abstract

**Background:**

The Toto Bora trial tested whether a 5-day course of azithromycin reduced the risk of rehospitalization or death in the 6 months following hospitalization among Kenyan children and found no overall benefit. We hypothesized that macrolide resistance in gut microbes could modify azithromycin's effect.

**Methods:**

From June 2016 to November 2019, Kenyan children aged 1–59 months were enrolled at hospital discharge and randomized to azithromycin or placebo. DNA from fecal samples and *Escherichia coli* isolates was analyzed for common macrolide resistance genes. Cox proportional hazards regression models, including interaction terms between randomization arm and individual macrolide resistance genes, were used to analyze time to rehospitalization or death, with Bonferroni correction applied to account for multiple comparisons.

**Results:**

Among 1393 children tested, 94.7% had at least 1 macrolide resistance gene in their fecal DNA at hospital discharge, most commonly *mph(A)* (68.6% [955/1393]), followed by *msr(D)* (67.3% [937/1393]) and *erm(B)* (60.7% [846/1393]). *Mef(A)* (23.7% [330/1393]) was the only macrolide resistance gene that modified azithromycin's effect on rehospitalization or death (interaction *P* = .008). In children without the *mef(A)* gene, azithromycin reduced the hazard of rehospitalization or death by a third (hazard ratio [HR], 0.66 [95% confidence interval {CI}, .45–.99]) whereas among children with the *mef(A)* gene, there was a higher risk in those randomized to azithromycin (HR, 2.72 [95% CI, 1.21–6.09]). The effect size of azithromycin's impact on mortality and rehospitalization as separate outcomes in children with and without *mef(A)* were consistent but underpowered.

**Conclusions:**

Macrolide resistance in the gut microbiome may influence the efficacy of azithromycin in children discharged from the hospital.

**Clinical Trials Registration.** NCT02414399.

## Teaser

The Toto Bora trial showed no overall benefit of azithromycin posthospitalization in Kenyan children. High prevalence of gut macrolide resistance was found. The *mef(A)* gene significantly modified azithromycin's effect. Gut microbiome resistance may influence azithromycin efficacy following hospital discharge.

More than 3 million children die each year from infectious causes, the majority of which (∼70%) are children living in sub-Saharan Africa [1]. Hospitalized children are at high risk of death during the acute episode and in the months following discharge and are particularly prone to new or sustained bacterial infections extending into the discharge period [2]. Based on cluster randomized trial evidence of mortality prevention observed with azithromycin mass drug administration (MDA) in high-mortality settings in sub-Saharan Africa and associated World Health Organization (WHO) guidelines [3–5], we conducted the Toto Bora trial to test whether a 5-day course of azithromycin delivered at hospital discharge prevented hospitalization and death in Kenyan children [6]. This double-blind, individually randomized, placebo-controlled trial found no evidence of benefit, consistent with the heterogeneity observed in MDA trials of azithromycin, which found geographic and age heterogeneity in its mortality-preventing effects [5, 7], and lack of benefit in an individually randomized trial of azithromycin delivered at routine infant health visits [8].

The precise mechanisms by which empiric azithromycin reduces childhood mortality remain unclear. Community-level MDA of azithromycin may exert herd effects such as reduced bacterial pathogen transmission that may not be exerted in individual-level trials of azithromycin. A substudy identifying a reduction in *Shigella* infection [9], along with verbal autopsy evidence of a reduction in dysentery deaths in MDA-treated communities [10], potentially implicates the gut, specifically enteric bacteria, as a contributor to azithromycin's effect. The gut microbiome comprises hundreds of bacteria, some of which may harbor antibiotic-resistant genes [11]. Without antibiotic treatment, these genes may have no clinical implications. However, in the presence of antibiotic treatment, such as empiric azithromycin, these genes may modify azithromycin's effect if this broad-spectrum antibiotic works partially via mechanisms involving the gut microbiome. Higher levels of macrolide resistance in the gut microbiota of children have been observed in communities exposed to MDA azithromycin [12–15].

Here, we sought to understand the implications of genotypic macrolide resistance in the gut microbiota (resistome) on azithromycin efficacy by testing fecal samples and *Escherichia coli* isolates for antibiotic resistance genes and evaluating the impact of azithromycin on death, rehospitalization, and linear growth among Kenyan children enrolled in the Toto Bora trial.

## METHODS

### Ethical Statement

The Toto Bora trial [16] was conducted in western Kenya with ethical approval from the Kenya Medical Research Institute (KEMRI) Scientific and Ethics Review Unit (SERU 3086, 8 September 2015), the Kenya Pharmacy and Poisons Board (ECCT/15/10/04, 3 December 2015), and the University of Washington Institutional Review Board (IRB 49120, 2 June 2015). All participants provided written informed consent to participate in the study and provided fecal samples for antimicrobial resistance (AMR) laboratory analyses at KEMRI research laboratories. The study was registered at ClinicalTrials.gov (NCT02414399) and conducted according to Good Clinical Practice guidelines and following the principles of the Declaration of Helsinki.

### Toto Bora Trial

The Toto Bota trial took place between 2016 and 2019. Full details of the study design and conduct are provided in the study protocol [16], and the main study results have been published previously [6]. At the point of hospital discharge, participants were randomly assigned 1:1 to receive either a 5-day course of azithromycin (oral suspension 10 mg/kg on day 1, followed by 5 mg/kg per day on days 2–5) or placebo. Eligibility criteria included children who were 1–59 months of age at the time of hospital discharge, weighed ≥2 kg, and had been hospitalized for any other medical cause except trauma, poisoning, or congenital anomaly. We excluded participants who had been prescribed a macrolide antibiotic at discharge or were taking a protease inhibitor for human immunodeficiency virus (HIV) infection, as well as concurrently enrolled same-sex twins or participants who intended to relocate out of the study area before the completion of the follow-up period [16].

### Study Sites

The trial was conducted in 3 hospitals within Homa Bay County (Homa Bay Teaching and Referral Hospital, St Paul Mission Hospital, and Kendu Adventist Mission Hospital) and the Kisii Teaching and Referral Hospital in Kisii County, Kenya. The Homa Bay County sites serve a predominantly rural/periurban population, while the Kisii site serves a predominantly urban population. The hospitals were targeted because they provide inpatient care and are located in regions with high child mortality rates. Further details of the study sites are provided in the protocol [16].

### Sample Collection and Laboratory Analysis

We collected distinct whole stool samples where possible, or rectal swabs using pediatric flocked swab (FLOQSwabs Self Collection; Copan) kits before azithromycin was administered. These specimens were immediately (within an hour) placed in cool boxes and maintained between 2°C and 8°C until arriving at the KEMRI Centre for Microbiology laboratory in Nairobi within 24 hours of collection for −80°C storage. As previously described [6], *E coli* was isolated by microbiologic culture using standard methods from distinct fecal samples.

### Nucleic Acid Extraction and Quantitative Polymerase Chain Reaction Using the TaqMan Array Card

Fecal samples and *E coli* isolates underwent quantitative polymerase chain reaction (PCR) testing using a TaqMan array card, using validated pathogen and antibiotic resistance probes and primers, between 2022 and 2023 at the KEMRI Centre for Global Health Research in Kisumu, Kenya. The total nucleic acid (TNA) material was extracted using the QIAamp Fast DNA Stool Mini Kit according to the manufacturer's instructions (Qiagen, Hilden, Germany) with a few modifications. In brief, rectal swabs or whole stool samples were combined with InhibitEX buffer (Qiagen) and glass beads (212–300 μm, Sigma) for 2 minutes of bead beating followed by 5 minutes of incubation at 95°C. After centrifugation at 20 000*g* for 1 minute, proteinase K (25 μL) was added to the supernatant along with AL buffer (600 μL), and the mixture was incubated at 70°C for 10 minutes. Ethanol (600 μL) was added and the lysate processed using QIAamp columns through centrifugation at 20 000*g*. Wash and elution buffers were applied to obtain TNA. The TNA was aliquoted into 2 Eppendorf tubes for storage at −80°C. Bacteriophage MS2 and phocine herpesvirus were included as controls for nucleic acid extraction and amplification.

Extracted DNA (∼40 μL) was combined with 50 μL Ag-Path-ID 2X RT-PCR buffer (Ambion), 4 μL Ag-Path-ID enzyme mix (containing reverse transcriptase and Taq polymerase enzymes), and 6 μL diethyl pyrocarbonate-treated water. This mixture was loaded into each port on the card and centrifuged at 1200 rpm twice for 1 minute each. The cards were then placed into the ViiA7 platform (Applied Biosystems, Thermo Fisher Scientific) with cycling conditions including reverse transcription at 45°C for 20 minutes and initial denaturation at 95°C for 10 minutes, followed by 40 cycles of denaturation at 95°C for 15 seconds and annealing/extension at 60°C for 1 minute. The cycle threshold (Ct) needed to detect a signal from the sample for each gene was recorded in a password-protected database (Multi-Schema Information Capture [MuSIC]) jointly managed by the University of Virginia and the collaborating local laboratory.

### Outcomes

The primary outcome in the parent trial was death or rehospitalization during the 6 months following hospital discharge. Deaths and rehospitalizations that occurred within the study sites were systematically recorded, including the cause of death and hospitalization, by trained study clinicians. Deaths occurring outside the hospital premises underwent a rigorous documentation process, involving the acquisition of official death certificates from family members or community leaders. This was supplemented by scrutiny of clinic records and verbal autopsies to elicit pertinent information regarding the circumstances surrounding the death. Hospital records were obtained for hospitalizations outside study sites where possible and we relied on caregiver reports when not available. Recumbent length (for children aged <24 months or unable to stand) and standing height (for those aged ≥24 months who could stand independently) were measured to the nearest 0.1 cm using a ShorrBoard at enrollment and the 3- and 6-month follow-up visits. Height-for-age/length-for-age z-scores were calculated for each child at each timepoint using the 2006 WHO growth standards for children <5 years of age [17].

### Data Analysis

A sample size of 1400 participants (700 per treatment arm) was sufficient for the primary clinical trial reported elsewhere [6, 16]. In this study, we included all children enrolled in the clinical trial that investigated the effect of a 5-day administration of azithromycin on postdischarge mortality or rehospitalization [6]. We evaluated the effect of azithromycin in children with or without each macrolide resistance gene—*erm(A)*, *erm(B)*, *erm(C)*, *mef(A)*, *mph(A)*, *mph(B)*, *msr(A)*, and *msr(D)*—detected in baseline fecal samples (primary) and *E coli* isolates (secondary). To test whether any effects were specific to macrolide resistance versus any resistance, we also analyzed *bla_CTX-M_* (the globally predominant and clinically significant extended-spectrum β-lactamase gene [18]) for comparison. We defined AMR gene positivity using a Ct value <30 to exclude detections of low gene quantity but repeated analyses using the lower limit of detection (Ct <35) as a sensitivity analysis. Cox proportional hazards regression models were used to assess time to rehospitalization or death, including an interaction term between the randomization arm and the presence/absence of the individual macrolide resistance genes. Hazard ratios (HRs) were estimated for the presence or absence of AMR genes and presented in forest plots to aid visual inspection. In addition, we investigated the potential effect modification of macrolide genes on azithromycin for improvement of linear growth among children who were alive at the end of the follow-up period using the linear mixed-effects model. The significance of the interaction terms was evaluated using the likelihood ratio test with Bonferroni-adjusted *P* values (adjusted for 7 tests) to correct for multiple testing. All statistical analyses were 2-sided, with Bonferroni-adjusted *P* values <.05 deemed statistically significant. All statistical analyses were performed in R version 4.4.1 and Stata version 18 (StataCorp) software.

## RESULTS

**Figure 1.**
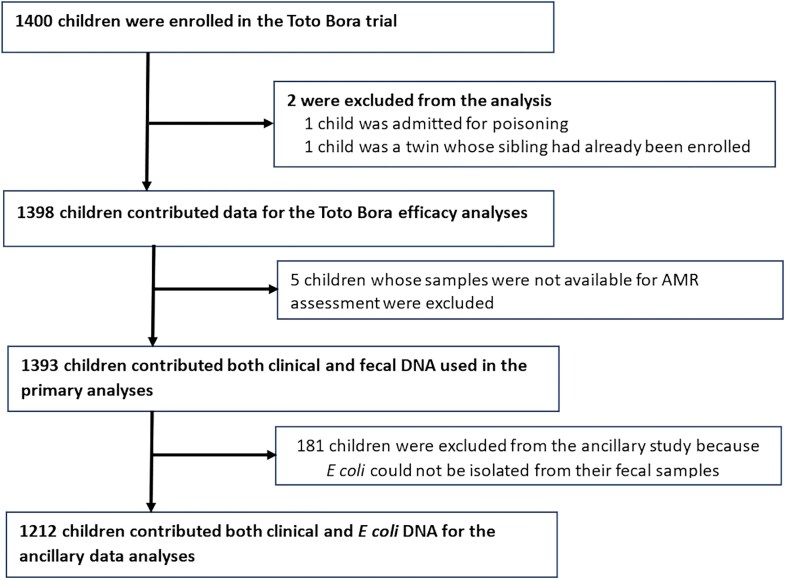
Study profile and reasons for exclusion at each stage. Abbreviation: AMR, antimicrobial resistance.

The baseline characteristics of study participants are described elsewhere [6]. In summary, between 28 June 2016 and 4 November 2019, 1400 children were enrolled in the Toto Bora trial: 703 (50.2%) received azithromycin, and 697 (49.8%) received a placebo. Two participants were withdrawn due to eligibility concerns after enrollment, and 5 children did not have sufficient fecal samples for laboratory analyses. Therefore, we analyzed data from 1393 children, of whom 699 (50.2%) received azithromycin and 696 (49.8%) received a placebo. Detailed descriptions of the reasons for exclusion at each stage for the parent trial have been published previously [6], and for this nested analysis are presented in Figure 1.

The median age at enrollment was 18 months (interquartile range [IQR], 9–32 months), and 570 (40.9%) were female.Enrollment sites were Kisii Teaching and Referral Hospital (818 participants [58.7%]), Homa Bay Teaching and Referral Hospital (520 participants [37.3%]), and St Paul Mission Hospital or Kendu Adventist Mission Hospital (55 participants [3.9%]). Among these participants, 75 (5.4%) had moderate wasting, 57 (4.1%) had severe wasting or edema, 313 (22.6%) were stunted, 175 (12.6%) were underweight, 18 (1.3%) had HIV infection, 147 (10.5%) were exposed to HIV, and 643 (46.4%) had received all age-appropriate vaccines. During hospitalization, 89.6% (n = 1248) of the children received antibiotics, with a median hospital stay of 3 days (IQR, 2–5 days). Common discharge diagnoses were lower respiratory tract infections (32.8%), malaria (25.1%), gastroenteritis or diarrhea (19.0%), and anemia (13.4%).

**Figure 2.**
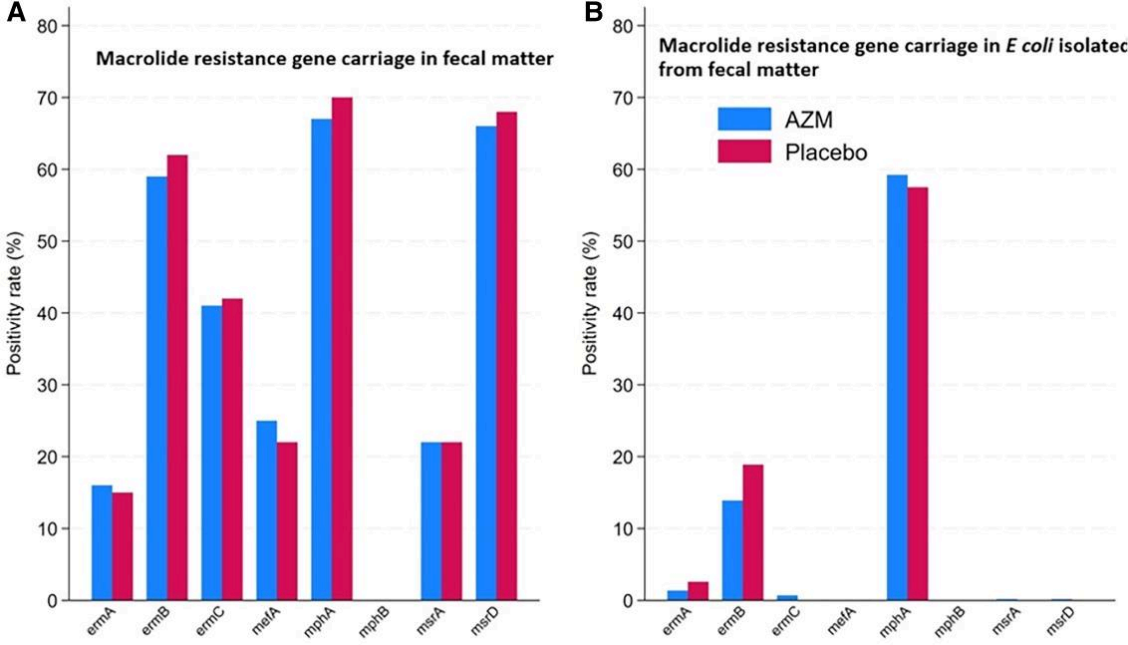
Distribution of macrolide resistance gene carriage at baseline by randomization arm (defined by cycle threshold <30). *A* and *B*, Antimicrobial resistance genes detected in fecal DNA (*A*) and *Escherichia coli* isolates (*B*).

**Figure 3.**
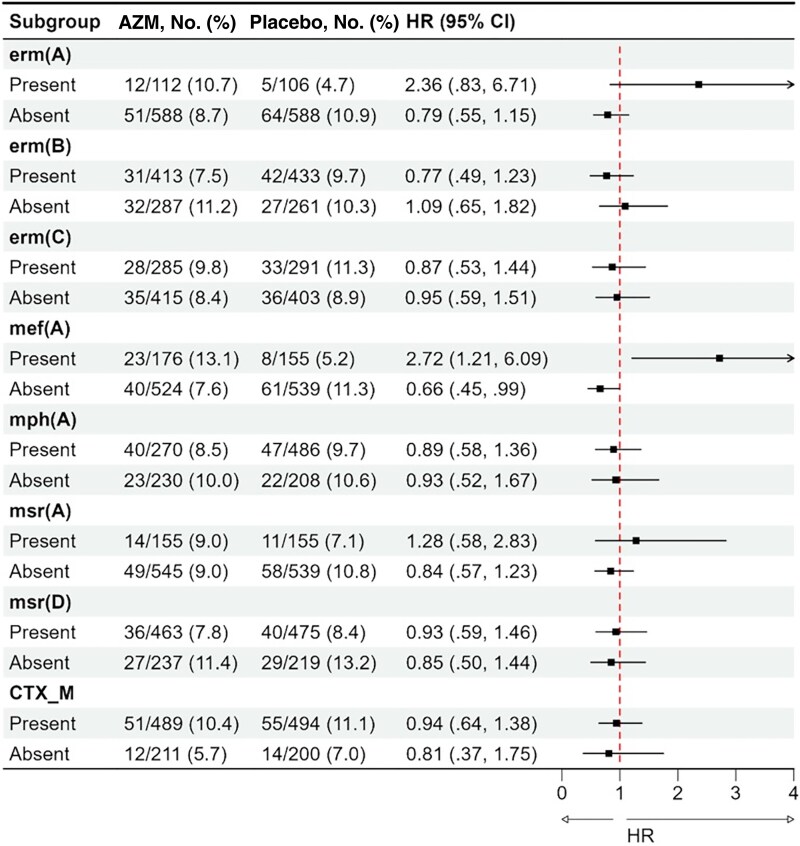
Effect of azithromycin (AZM) for preventing rehospitalization or death (composite outcome) among children discharged from hospital with and without the specified antimicrobial resistance gene in fecal DNA (defined by cycle threshold <30). The black square dots and the error bars represent the hazard ratio (HR) and 95% confidence interval (CI).

Most children (94.7%) had at least 1 macrolide resistance–conferring gene detected in their discharge fecal sample. The macrolide gene carriage distribution was similar across the randomization arms in fecal samples (Figure 2*A*) and *E coli* isolates (Figure 2*B*). Overall, 34 (2.4%) children died within the 180 days following hospital discharge, and 114 (8.2%) children were hospitalized at least once, leading to a combined incidence rate of 20.2 per 100 child-years in the azithromycin arm and 22.6 per 100 child-years in the placebo arm. Among children without the *mef(A)* gene in fecal DNA, the incidence of the combined outcome of death or first rehospitalization was 17.2 per 100 child-years in children randomly assigned to azithromycin and 25.9 per 100 child-years in the placebo group (HR, 0.66 [95% confidence interval {CI}, .45–.99]; *P* = .044). Among children with the *mef(A)* gene, the incidence of the combined outcome of death or first rehospitalization was 29.5 per 100 child-years in children randomly assigned to azithromycin and 11.4 per 100 child-years in the placebo group (HR, 2.72 [95% CI, 1.21–6.09]; *P* = .015). There was statistical evidence of the effect modification of azithromycin by *mef(A)* (interaction term likelihood ratio test, Bonferroni-adjusted *P* = .008) (Figure 3). Similar effect size differences between those with and without the *mef(A)* gene were observed when considering the 2 outcomes individually (Supplementary Figures 1 and 2), and the likelihood ratio test for interaction adjusted *P* values were .061 and .056 for death and rehospitalization, respectively. There was no evidence of the effect modification of azithromycin by macrolide resistance–conferring genes in *E coli* isolated from children at the same time point (Supplementary Figure 3).

**Figure 4.**
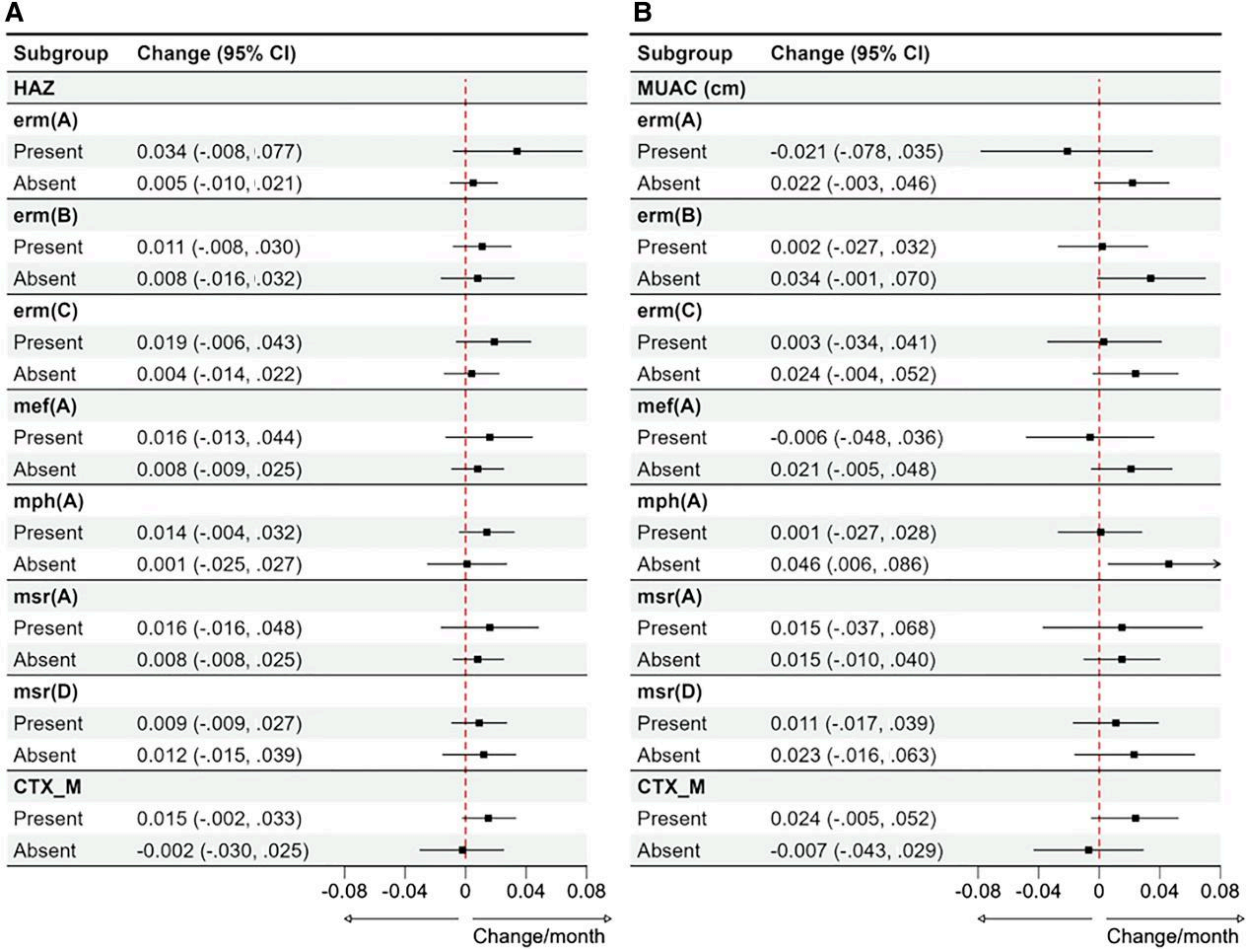
Effect of azithromycin on change in length-for-age/height-for-age z-score (HAZ, *A*) and mid-upper arm circumference (MUAC, *B*) between enrollment and 6-month follow-up among children discharged from hospital with and without the specified antimicrobial resistance gene detected in fecal DNA (defined by cycle threshold <30). Data represent the monthly change in HAZ and MUAC (cm), respectively. The black square dots and the error bars represent the net monthly change in growth between the azithromycin group and the placebo group, with 95% confidence intervals (CIs).

There were no major differences in azithromycin effect across other macrolide resistance genes detected in fecal DNA (Figure 3, Supplementary Figures 1 and 2), and the effect of azithromycin on rehospitalization or death did not appear to be modified by *bla_CTX-M_* (HR, 0.94 [95% CI, .64–1.38] for presence of *bla_CTX-M_* vs 0.81 [95% CI, .37–1.75] for absence of *bla_CTX-M_*; likelihood ratio test for interaction adjusted *P* >.99). In addition, there was no evidence of an effect of azithromycin on linear growth across the macrolide genes in fecal DNA (Figure 4), nor in *E coli* isolates (Supplementary Figure 4). Our findings remained consistent in a sensitivity analysis using a threshold of Ct <35 to evaluate the impact of including lower gene quantities (Supplementary Figures 5–7).

## DISCUSSION

In this study, we explored potential gut-mediated mechanisms explaining a lack of individual-level treatment effect of azithromycin for preventing mortality and rehospitalization among Kenyan children discharged from inpatient care. We found evidence that 5-day treatment with azithromycin was associated with a 35% decrease in the risk of rehospitalization or death among children without the *mef(A)* macrolide resistance–conferring gene. Conversely, among children with the *mef(A)* macrolide gene, azithromycin was associated with an approximately 3-fold increase in the risk of rehospitalization or death. These findings were consistent when each outcome (mortality or death) was examined separately, albeit with low statistical power. However, we did not observe effect modification by other macrolide resistance–conferring genes, such as *mph(A)*, despite being commonly detected.

The *mef(A)* gene encodes the transmembrane channel component of an ATP-binding cassette transporter that confers macrolide resistance [19]. It has primarily been studied in gram-positive bacteria, in particular streptococci [20], which are a prominent constituent of the gut microbiota in young children with diarrhea in low-income countries [21]. Gram-negative bacteria such as *Shigella* may also carry *mef(A)* [22], and *Shigella* has been implicated in azithromycin effect mechanisms [9]. However, the lack of effect modification by *mef(A)* in *E coli*, also a member of the Enterobacteriaceae family, suggests that this may not be the case. The expansion of macrolide-resistant pathogens within the microbiota may explain the adverse impact of azithromycin on *mef(A)*-positive individuals. Further, if treatment of gram-positive pathogens is a key mechanism of azithromycin efficacy during the postdischarge period, it follows that we observed a treatment benefit among children without evidence of *mef(A)* resistance.

Approximately 95% of the children had at least 1 macrolide resistance–conferring gene in their gut microbiome at discharge. That we did not observe similar effect modification by other macrolide resistance–conferring genes, such as the commonly reported *mph(A)* gene, is noteworthy. It could be that pathogens uniquely affected by *mef(A)*, such as streptococci, are responsible for the higher risk of death and rehospitalization in this postdischarge population. Unfortunately, this study could not identify the bacterial species harboring *mef(A)* to probe this explanation. We also cannot exclude the possibility that these findings represent a type I error. Further studies are needed to validate these findings that a specific macrolide resistance–conferring gene may influence azithromycin's effect at an individual level.

We have previously characterized phenotypic and molecular determinants of macrolide and β-lactam resistance carriage among *E coli* and *Klebsiella* isolates from a subset of children enrolled in the Toto Bora trial and observed a high degree of phenotypic and genotypic resistance [23, 24]. The high prevalence of macrolide resistance genes, likely reflecting high antibiotic use in hospitals as well as community settings, poses an important limitation. This may restrict the generalizability of our findings to settings with similar resistance rates. Also, the high amount of antibiotic exposure before or concurrently with azithromycin introduces challenges with generalizability.

We saw no impact of azithromycin on linear growth overall [25], nor when stratifying by macrolide resistance genes in this study. Azithromycin has been demonstrated to offer growth-promoting benefits when targeted to children with diarrhea attributed to specific enteric bacteria [26], but similar findings have not been found in MDA trials [27, 28]. This antibiotic has shown efficacy in reducing the incidence of infectious diseases, which can indirectly support better growth and development in children [26]. However, the potential use of this broad-spectrum antibiotic for multicausal and long-term processes like linear growth raises concerns about antibiotic resistance [14, 15].

Our study is the first to systematically evaluate the potentially modifiable effect of macrolide resistance gene carriage on the efficacy of azithromycin for posthospital rehospitalization and mortality. If confirmed, this finding could explain sources of variability in treatment effects in empiric azithromycin trials targeting children at high risk of mortality. In the long term, identifying a biomarker predictive of treatment efficacy could result in fewer children being exposed to empiric azithromycin if a point-of-care diagnostic could be developed, such as rifampicin resistance detection in GeneXpert tests for tuberculosis [29]. A limitation of our study is that although we identified certain macrolide resistance-conferring genes that altered the effect of azithromycin, the study was underpowered to detect variations in azithromycin's effect across subgroups.

Despite an overall reduction in child mortality, children recently discharged from hospital remain at an elevated risk of mortality and contribute to almost half of the total mortality rate in low- and middle-income countries [2]. Azithromycin appeared to have a benefit in a subset of children identified by a specific macrolide resistance gene, a finding that needs to be confirmed in other populations.

## Supplementary Material

jiaf208_Supplementary_Data

## Supplementary Data

Supplementary materials are available at *The Journal of Infectious Diseases* online (http://jid.oxfordjournals.org/). Supplementary materials consist of data provided by the author that are published to benefit the reader. The posted materials are not copyedited. The contents of all supplementary data are the sole responsibility of the authors. Questions or messages regarding errors should be addressed to the author.

## Notes

***Authors contributions.*** Conceptualization and design: J. B. O., B. O. S., J. L., J. A. P., F. C. F., J. L. W., E. R. H., and P. B. P. Performed the experiments: J. B. O., L. R. A., C. S., E. A., L. A., D. R., K. K., B. O. S., J. L., and P. B. P. Data curation: P. M., K. D. T., M. M. D., and J. L. Funding acquisition: J. L. W. and P. B. P. Project administration and investigation: H. A., K. D. T., M. D. D., B. O. S., J. L., and P. B. P. Formal data analysis: P. M. Writing–original draft: P. M. Writing–review and editing: All authors.

***Disclaimer.*** The funder had no role in data collection, analysis, or interpretation; writing of the report; or the decision to submit the manuscript for publication.

***Data availability.*** The datasets from the parent Toto Bora trial, linked with the antimicrobial resistance laboratory data utilized in these analyses, are publicly available on the Dryad repository (https://doi.org/10.5061/dryad.m0cfxppfv). The statistical analysis code used in this study is also published online alongside the data.

***Financial support.*** This work was supported by the Eunice Kennedy Shriver National Institute of Child Health and Human Development, National Institutes of Health (grant number R01HD079695 to J. L. W.); and the National Institute of Allergy and Infectious Diseases, National Institutes of Health (grant number 1R01AI150978 to P. B. P.).
