# Supplementary material for: Impact of Macrolide Resistance on Azithromycin for Prevention of Rehospitalization or Death Among Children Discharged From Hospitals in Western Kenya
Source: J Infect Dis. 2025 Apr 21;232(2):e301–8. doi: 10.1093/infdis/jiaf208 (PMC12349937; doi:10.1093/infdis/jiaf208)
Supplement: jiaf208_Supplementary_Data [file jiaf208_supplementary_data.docx]

**Supplementary Materials**

**
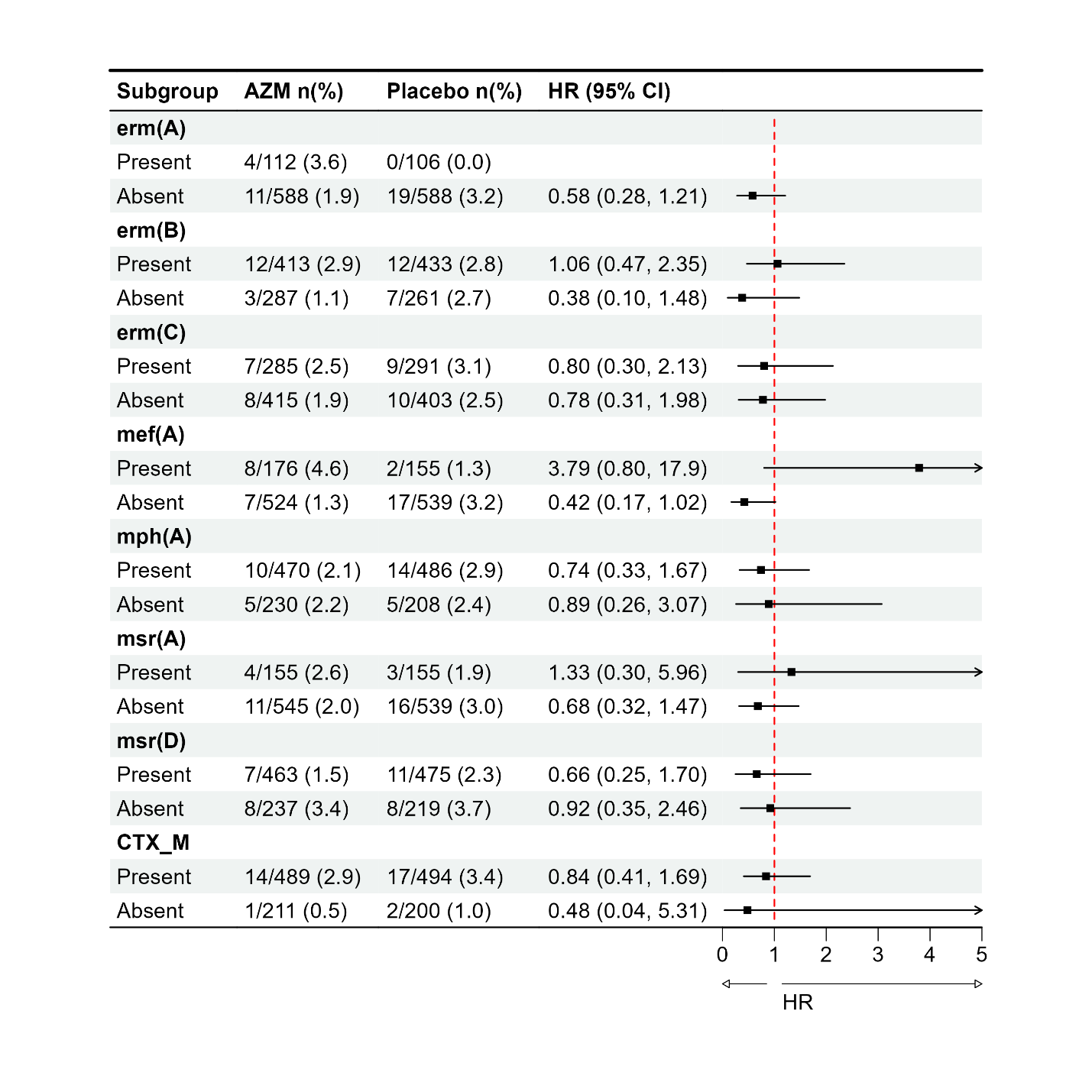
**

**Supplementary Figure 1: Effect of azithromycin for preventing death among children discharged from hospital with and without the specified AMR gene detected in fecal DNA (defined by CT<30)**. The black square dots and the error bars represent the hazard ratio (HR) and 95% confidence intervals.

**
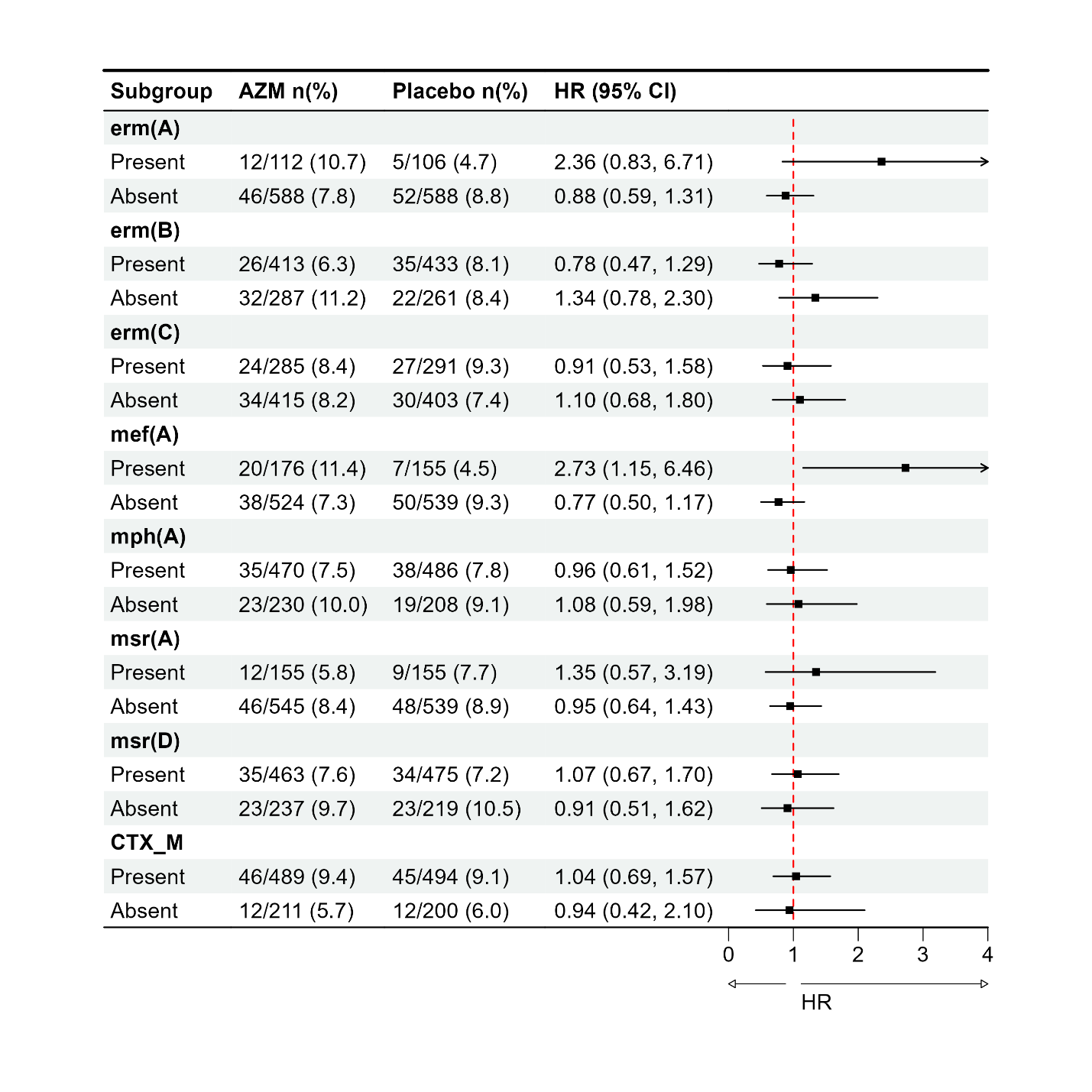
**

**Supplementary Figure 2: Effect of azithromycin for preventing hospitalization among children discharged from hospital with and without the specified AMR gene** **detected in fecal DNA (defined by CT<30)**. The black square dots and the error bars represent the hazard ratio (HR) and 95% confidence intervals.


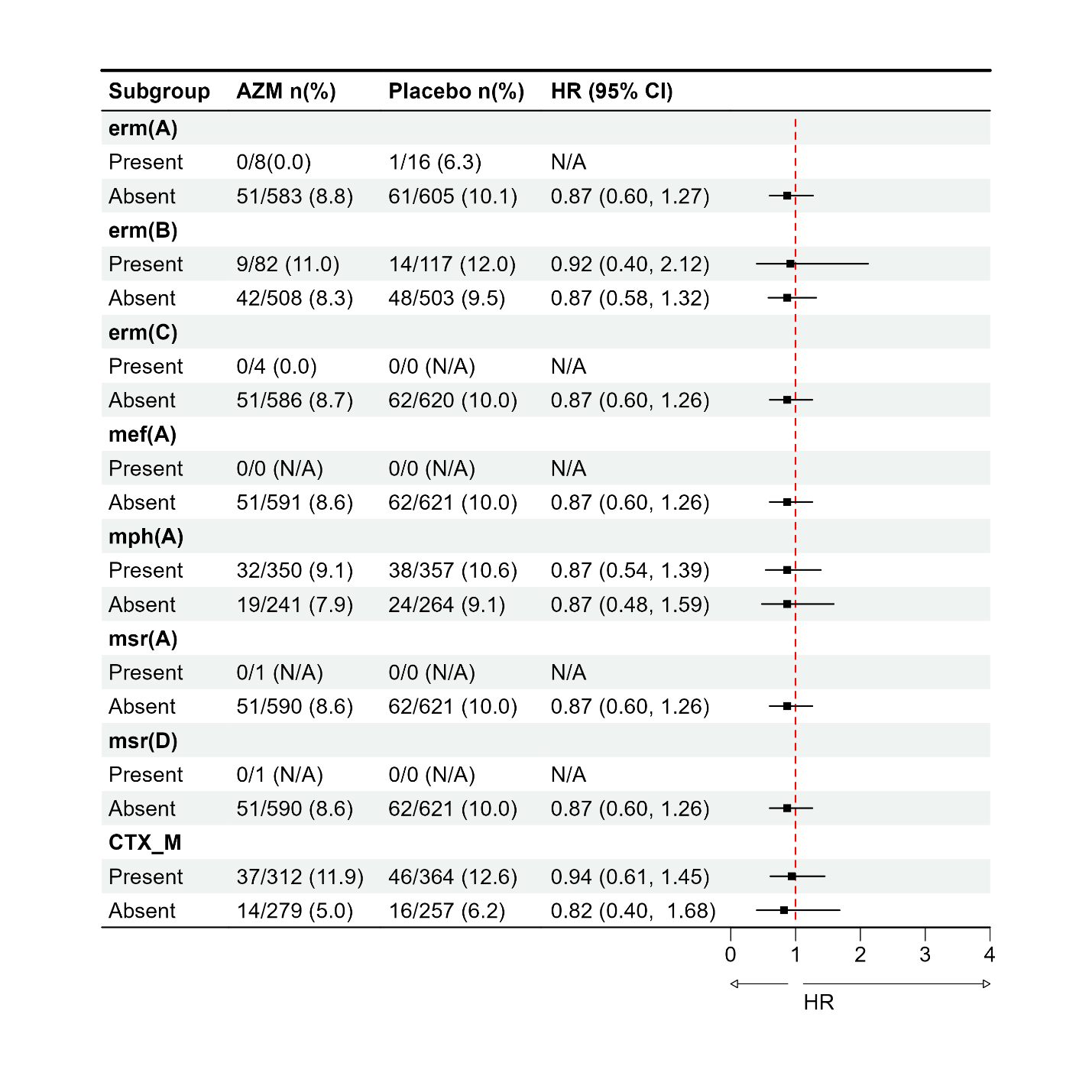


**Supplementary Figure 3: Effect of azithromycin for preventing rehospitalization or death (composite outcome) among children discharged from hospital with and without the specified AMR gene** **in *E. coli* isolates (defined by CT<30)**. The black square dots and the error bars represent the hazard ratio (HR) and 95% confidence intervals.

**
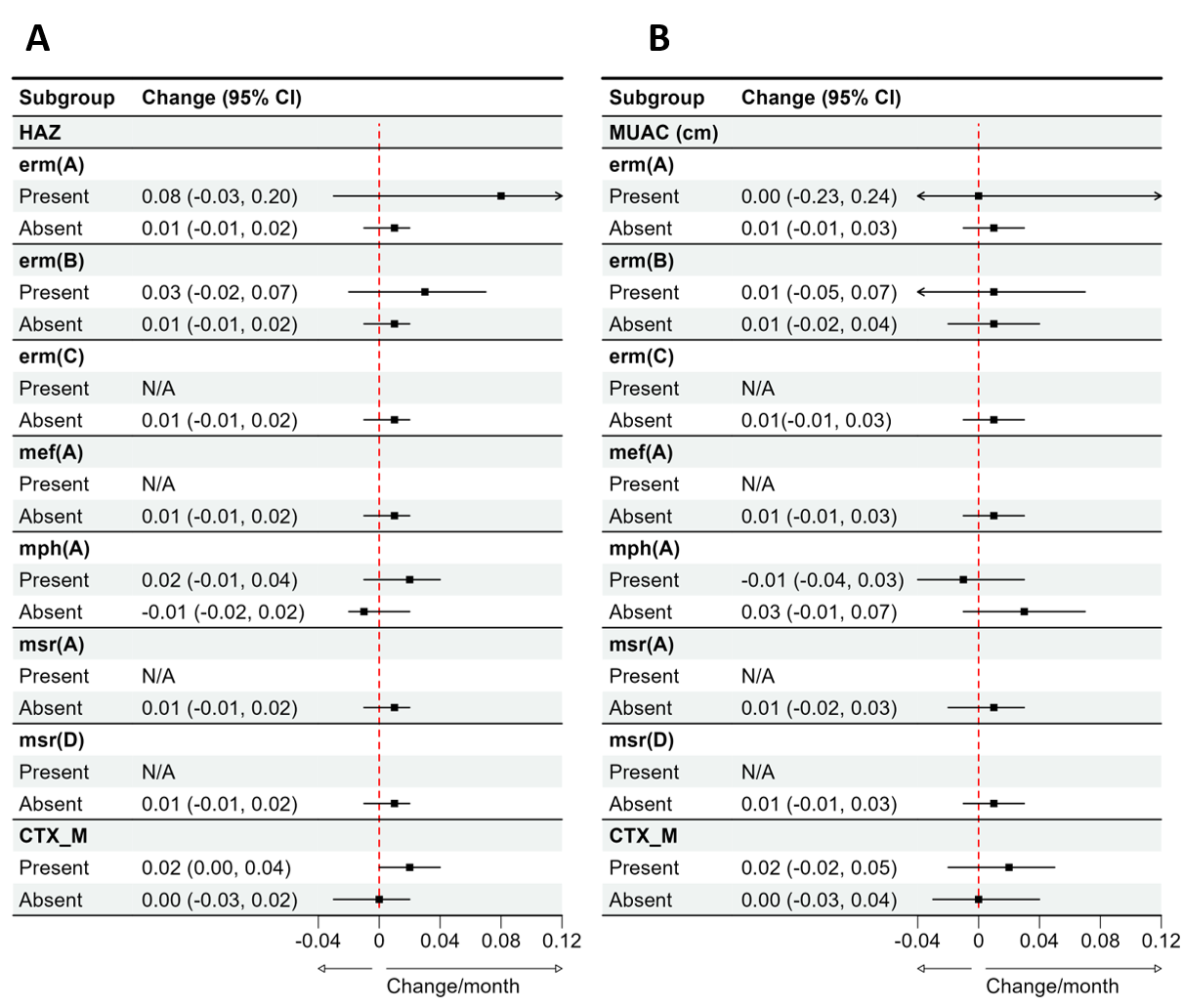
**

**Supplementary Figure 4: Effect of azithromycin on change in length/height-for-age z-score (LAZ/HAZ) and mid-upper arm circumference (MUAC) between enrollment and 6 months follow–up among children discharged from hospital with and without the specified AMR gene detected in *E coli* isolates (defined by CT<30)**. Panels A and B represent the monthly change in LAZ/HAZ and MUAC (cm) respectively. The black square dots and the error bars represent the net monthly change in growth between the azithromycin group and the placebo, and the 95% confidence intervals.


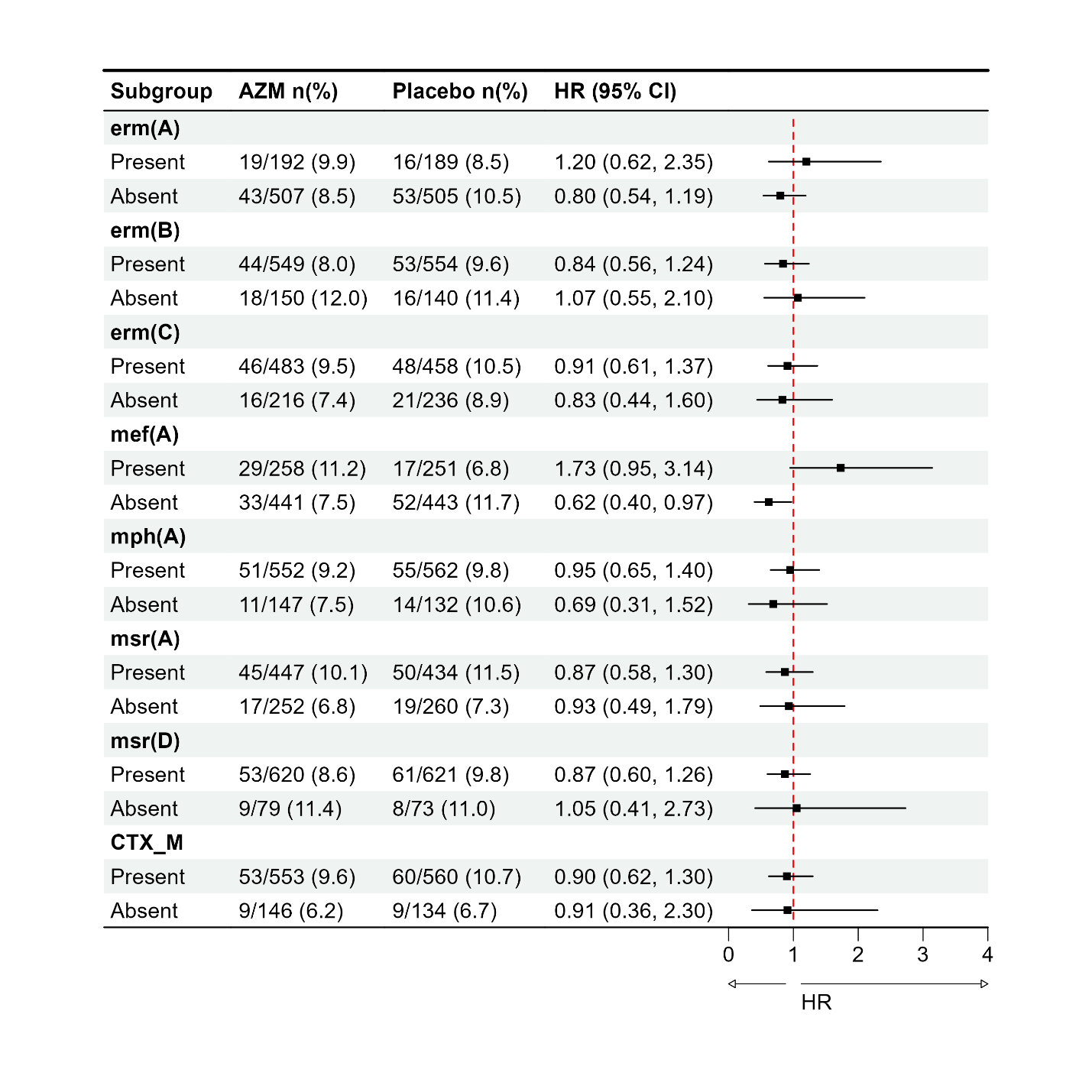


**Supplementary Figure 5: Effect of azithromycin for preventing rehospitalization or death (composite outcome) among children discharged from hospital with and without the specified AMR gene detected in fecal DNA (defined by CT<35)**. The black square dots and the error bars represent the hazard ratio (HR) and 95% confidence intervals.


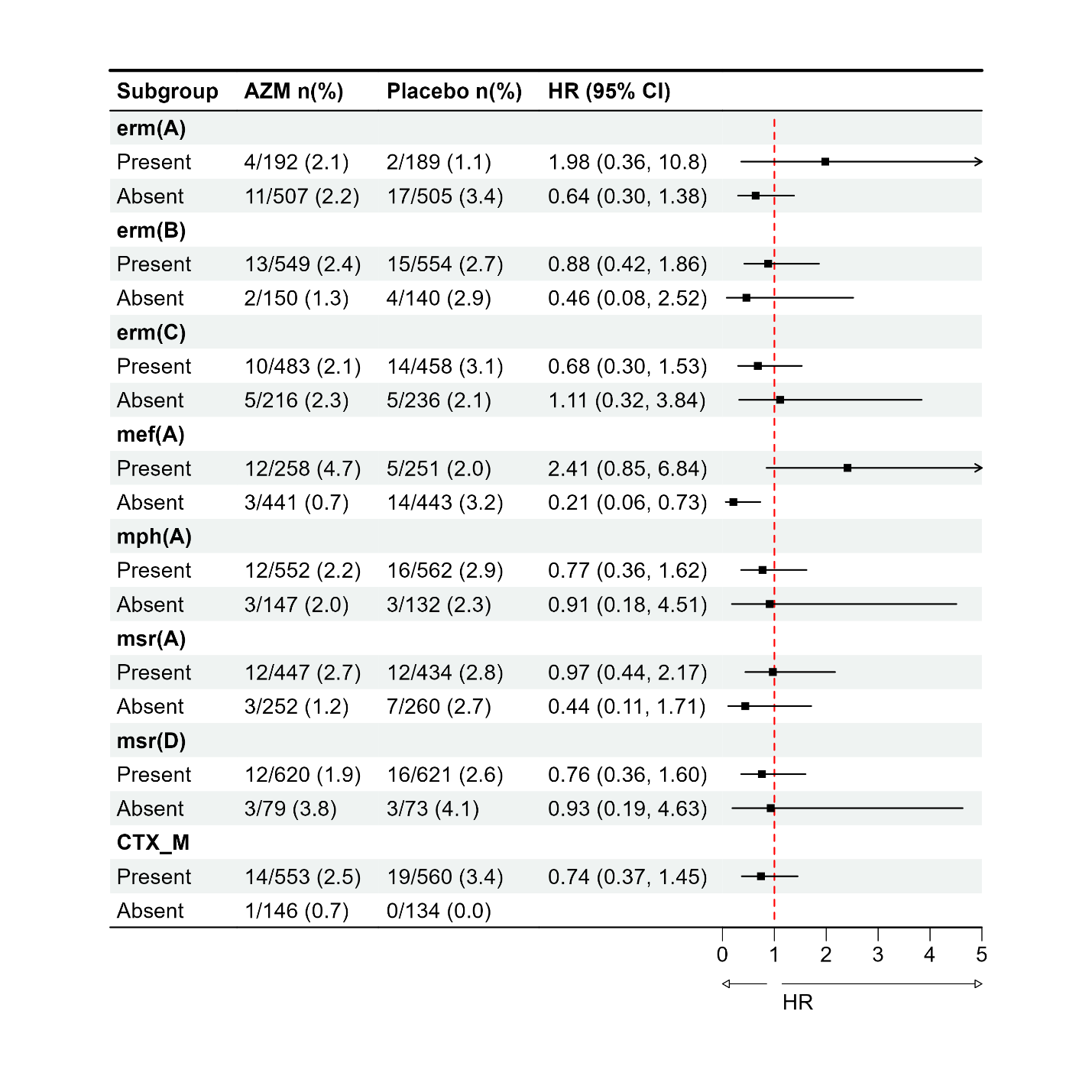


**Supplementary Figure 6: Effect of azithromycin for preventing death among children discharged from hospital with and without the specified AMR gene detected in fecal DNA (defined by CT<35)**. The black square dots and the error bars represent the hazard ratio (HR) and 95% confidence intervals.


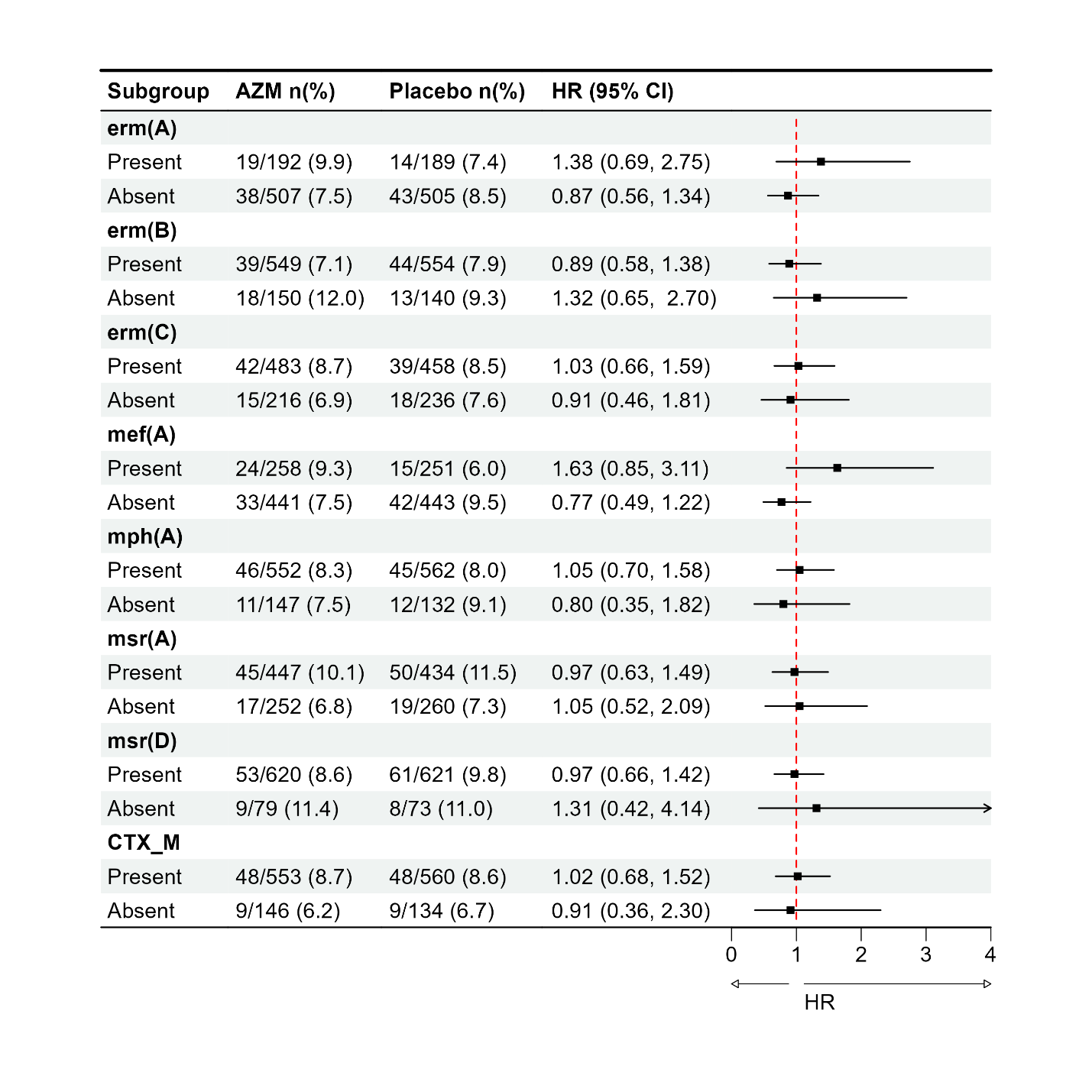


**Supplementary Figure 7: Effect of azithromycin for preventing rehospitalization among children discharged from hospital with and without the specified AMR gene detected in fecal DNA (defined by CT<35)**. The black square dots and the error bars represent the hazard ratio (HR) and 95% confidence intervals.
